# Reasons and risk factors for delayed discharge after day-surgery percutaneous nephrolithotomy

**DOI:** 10.1186/s12894-022-01159-5

**Published:** 2022-12-21

**Authors:** Huacai Zhu, Bangfeng Liu, Mehmet Ali Karagöz, Gaoyuanzhi Yue, Yeci Lei, Shangwen Dou, Zhanping Xu, Yongda Liu

**Affiliations:** 1 Department of Urology and Guangdong Key Laboratory of Urology, The First Affiliated Hospital of Guangzhou Medical University, Guangzhou, 510230 Guangdong China; 2 Department of Urology, Foshan Hospital of Traditional Chinese Medicine, Foshan, 528000 Guangdong China; 3 Department of Urology, Faculty of Medicine, Kafkas University, 36000 Kars, Turkey; 4 Minimally Invasive Surgery Center, The First Affiliated Hospital of Guangzhou Medical University, Kangda Road 1#, Haizhu District, Guangzhou, 510230 Guangdong China

**Keywords:** Percutaneous nephrolithotomy, Day-surgery, Delayed discharge, Risk factor, Complication

## Abstract

**Background:**

Day-surgery percutaneous nephrolithotomy (PCNL) is being developed quickly but some potential factors are affecting the recovery process. This study is aim to analyze the reasons and risk factors for delayed discharge after day-surgery PCNL.

**Methods:**

The data of 205 patients who accepted day-surgery PCNL in our institution between January 2018 and February 2020 were analyzed, retrospectively. Univariate and multivariate logistic regression analysis were used to analyze the risk factors for delayed discharge. Besides, the nomogram prediction model was established by the multivariable logistic regression analysis.

**Results:**

The rate of delayed discharge was 14.6%. Independent risk factors for delayed discharge were larger stone burden (odds ratio [OR] = 3.814, P = 0.046), positive urine nitrite (OR = 1.001, P = 0.030), longer duration of surgery (OR = 1.020, P = 0.044), multiple nephrostomy tubes (OR = 4.282, P = 0.008). The five main reasons that caused delayed discharge included psychological reasons, pain, bleeding, urosepsis, and urine leakage.

**Conclusions:**

This study identified some independent risk factors for a hospital length of stay longer than 24 h. Patients with larger renal stones or positive urine nitrite may be at increased risk of delayed discharge after day-surgery PCNL. Reducing surgery time and nephrostomy tubes will help to facilitate recovery.

## Background

Renal stone is a very common disease that affects 5.8% of the population in China [1]. Percutaneous nephrolithotomy (PCNL) is considered as the most efficient treatment for renal calculi that are ≥ 2 cm in diameter, providing high stone-free rate and rapid recovery [2, 3]. Since the concept of enhanced recovery after surgery (ERAS) was introduced, shorter length of hospital stay (LOS) and fewer complications have been required by urologists [4]. Day-surgery PCNL is just as safe and effective as inpatient PCNL, with a good level of perceived quality [5–9]. Both surgeons and patients are willing to accept day-surgery PCNL because it can facilitate recovery, reduce the healthcare burden, and optimize hospital resources [8].

The requirement for day-surgery PCNL will increase, and as hospitals confront the problems of cost containment, the significance of discharge within 24 h increases. Although the majority of patients recuperate rapidly and can be discharged on the same day or within 24 h, some patients still required a stay in hospital of more than one day after surgery due to slow recovery. It has been reported[5, 7, 9–11] that the rate of delayed discharge ranges from 0 to 34% and the main reasons for delayed discharge include psychological reasons, pain, bleeding, urinary tract infection (UTI), urosepsis, urinary leakage, pleural effusion and urinary retention. To date, few articles have reported the risk factors of delayed discharge after day-surgery percutaneous nephrolithotomy. When trying to shorten the LOS, distinguishing these patient subgroups who recover slowly ensures that the correct intervening measures are directed to the proper patients. Moreover, determining the most frequent causes for patients who failed to be discharged within 24 h, may provide a better comprehension of how to further shorten the LOS.

In the current study, we aim to identify the risk factors associated with delayed discharge after day-surgery PCNL, and evaluate the most frequent causes of prolonged LOS.

## Methods

Data were retrospectively gathered from 205 patients who accepted day-surgery PCNL between January 2018 and February 2020 in our institution. Approval for this study was granted by the Local Ethics Committee of the First Affiliated Hospital of Guangzhou Medical University. The approval number is medical research ethics 2022 No. K-24. Because of the retrospective nature of the study, written informed consent was not required. All the surgeries were operated by a senior urologist (YD L) who specialized in lithotripsy, and has the experience of more than 150 PCNL a year. Day-surgery PCNL was defined as discharging patients within 24 h after operation[5]. LOS was calculated by the number of hours between operation and discharge. Delayed discharge was defined as staying in hospital longer than 24 h. These patients were then compared to the patients with normal discharge time (within 24 h) using univariate and multivariate logistic regression analyses. Patients who stayed at ward for more than 24 h due to psychological reasons were defined as patients encountering discharge criteria but refusing to leave hospital because they feared the development of adverse events. The Clavien-Dindo classification was used to grade the postoperative complications[12]. The stone free rate (SFR) was defined as the status of either no residual stone or ≤ 3 mm residual stones[13]. Day-surgery PCNL were performed after acquiring patients’ permission and signing the related consent forms.

A protocol for day-surgery PCNL and ERAS has been used at our institution since 2015. All patients receive both oral and written information about how to prepare for the operation and what the course of recovery should be. The main exclusion criteria included: (1) American Society of Anesthesiologists grade (ASA) ≥ 3; (2) World Health Organization performance status ≥ 2; (3) uncontrolled urinary tract infection; (4) severe damage of renal function; and (5) rejection of day surgery. All patients who didn’t meet the exclusion criteria were eligible for day-surgery PCNL. These patients were assessed preoperatively in the Urology Department Outpatients Clinic. Preoperative routine tests were also applied in the clinic. The safety of general anesthesia was evaluated in all patients before surgery by anesthetists. Prophylactic antibiotics were administered routinely thirty minutes before and six hours after the operation. In addition, appropriate oral antibiotics would be administered at least one week before surgery if patients suffered from positive urine culture or nitrite (NIT). The main procedures of percutaneous nephrolithotomy were performed as described elsewhere[14]. When the patient was eligible for day-surgery PCNL, the ERAS program was implemented. Pre-operation consulting, preoperative preparation (fasting for solids within 6 h and liquids within 2 h before surgery, oral nutritional drink, and prophylactic antibiotics), and postoperative rehabilitation management (prophylactic pain control, early mobilization, early ingestion of oral fluids and solids, and early removal of nephrostomy tubes and urinary catheter) were included in the ERAS protocol. Analgesia was routinely used in all patients after surgery to relieve and prevent pain.

Most patients could leave hospital on the same day after 4–6 h of rehabilitation in the day-surgery unit. If the patient’s permanent residence was not in the same province as our hospital, it was suggested that they could stay overnight. In addition, patients with surgery finishing time after 4:00 p.m. also stayed in hospital overnight to control and notice the complications. The discharge criteria were as follows: (1) steady vital signs; (2) no complications or completely recovered complications; and (3) no discomfort or discomfort which could be controlled by drug therapy. All nephrostomy tubes and urinary catheters were removed from the patients prior to discharge. The criteria of removing nephrostomy tubes were as follows: (1) steady vital signs; (2) the color of drainage fluid was clear or light red; (3) no obvious discomfort after the clamping of nephrostomy tubes for 1 h; (4) no symptoms of infection such as fever; (5) no second-stage PCNL was planned. Contact information of the hospital and the surgeon was given to day-surgery patients for the purpose of obtaining timely medical care in case of complications or problems developed at home. A non-contrast CT scan was used to assess the stone-free status after two weeks postoperatively. If the non-contrast CT scan could not be performed due to patients’ personal reasons, the KUB was used. Then the double-J stents were removed.

Forming retrospective examination of the medical records of patients, the major reasons for delayed discharge were summarized. The basic patient data, the operating notes and data of perioperative examinations were retrieved from the institutional database. The following variables were noted: LOS, gender, age, body mass index (BMI), comorbidity, same side previous urological procedure history, urine leukocyte (U-LEU), NIT, midstream urine culture, stone size, stone density, grade of hydronephrosis, stone type, surgery time, number of tracts, diameter of sheath, number of nephrostomy tubes and SFR.

### Statistical analysis

Data obtained in the study were analyzed using SPSS vn. 23 software. Continuous variables were compared using the Student’s t-test or Mann–Whitney u test. Binary variables were applied the chi-square test and ordinal variables were used Wilcoxon rank sum test to compare the difference between the two groups. The risk factors were analyzed by univariate and multivariate logistic regression analysis. First, univariate logistic regression was performed for each variable and those with a p-value of < 0.10 were included in the multivariate logistic regression analysis. The multivariate model was set up by a stepwise forward procedure (inclusion criteria was P < 0.05). A p-value < 0.05 was regarded as statistical significance.

Nomogram for delayed discharge was established by the R software (version R 3.6.1) rms package based on the results of multivariate analysis. The internal calibration of nomogram was assessed by calibration plot. The concordance index (c-index) was used to evaluate the discriminative ability of nomogram.

## Results

### Clinical and perioperative data

The surgical records of 205 patients were reviewed. The demographic characteristics of the patients and the perioperative findings are showed in Table 1. The average age was 51.7 years, and 126 (61.5%) patients were male. LOS ≤ 24 h was recorded for 175 (85.4%) patients (same-day discharge for 142 cases and overnight-stay discharge for 33 cases), and LOS > 24 h for 30 (14.6%). Two-week SFR was 88.8%. Compared with the group of LOS ≤ 24 h, the delayed discharge group had more patients with positive U-LEU (P < 0.005) and NIT (P < 0.009). Female patients had a higher incidence of delayed discharge than male patients (P < 0.027), among patients with longer operation time, larger stone burden, staghorn stone, larger tract, multiple nephrostomy tubes, the placement of nephrostomy tubes after surgery were higher in the delayed discharge group than in the control group (P < 0.05). In addition, there were no significant differences in age, BMI, comorbidity, history of previous ipsilateral stone surgery, urine culture, degree of hydronephrosis, stone density and SFR between the two groups (P > 0.05).

**Table 1:** Comparison of clinical data between patients with and without delayed discharge after day-surgery PCNL

Variable	Cohort (n = 205)	LOS^a^ ≤ 24 h (n = 175)	LOS^a^ > 24 (n = 30)	P-Value
Age (y)	51.7 ± 12.4	51.7 ± 12.4	51.6 ± 12.6	0.967
Gender				0.027
Male	126	113	13	
Female	79	62	17	
BMI (kg/m^2^)	23.4 ± 3.3	23.6 ± 3.3	22.5 ± 3.0	0.103
Comorbidity				1.000
No	173	147	26	
Yes	32	28	4	
History of previous ipsilateral stone surgery				
No	177	153	24	0.216
Yes	28	22	6	
U-LEU				0.005
−	153	137	16	
+	24	17	7	
+ +	18	14	4	
+ + +	8	5	3	
+ + + +	2	2	0	
NIT				0.009
−	177	156	21	
+	28	19	9	
Urine culture				0.273
−	173	150	23	
+	32	25	7	
Stone burden (mm^2^)	631.5 ± 644.4	543.5 ± 589.4	1144.6 ± 719.5	< 0.001
Stone density (HU)	956.4 ± 272.7	956.1 ± 271.0	958.3 ± 287.3	0.966
Degree of hydronephrosis 0.755				
None	31	26	5	
Mild	76	65	11	
Moderate	58	49	9	
Serious	40	35	5	
Stone type				0.051
Single	29	27	2	
Multiple	104	91	13	
Staghorn	72	57	15	
Operative time (min)	74.6 ± 30.4	70.3 ± 25.2	99.7 ± 44.0	< 0.001
Number of tracts				< 0.001
1	145	134	11	
2	28	20	8	
3	14	11	3	
4–7	18	10	8	
Tract size				0.001
Standard	38	26	12	
Mini	167	149	18	
Number of nephrostomy tubes < 0.001				
0	77	74	3	
1	89	78	11	
2	33	21	12	
3	5	2	3	
4	1	0	1	
SFR	88.8% (182/205)	90.3% (158/175)	80.0% (24/30)	0.117
Under CT	87.7% (128/146)	89.0% (113/127)	78.9% (15/19)	
Under X-ray	9 1.5% (54/59)	93.8% (45/48)	81.8% (9/11)	

PCNL, percutaneous nephrolithotomy; LOS, length of stay; BMI, body mass index; U-LEU, urine leukocyte; NIT, urine nitrite; SFR: stone free rate; CT: computed tomography

^a^LOS was counted as the number of hours between surgery and discharge

### Logistic regression analysis and risk factors

In the univariable logistic regression analysis (Table 2), the following variables were significantly associated with delayed discharge: gender (odds ratio [OR] = 2.383, P = 0.030), U-LEU (OR = 1.534, P = 0.023), NIT (OR = 3.519, P = 0.007), stone burden (OR = 1.001, P < 0.001), stone type (OR = 1.865, P = 0.051), surgery time (OR = 1.028, P < 0.001), number of nephrostomy tracts (OR = 2.074, P < 0.001), tract size (OR = 0.262, P = 0.002), and number of nephrostomy tubes (OR = 3.716, P < 0.001).

**Table 2:** Univariate and multivariate logistic regression analyses of risk factors for delayed discharge after day-surgery PCNL

Variables	Univariate analysis			Multivariate analysis		
	OR	95% CI	P value	OR	95% CI	P value
Age (y)	0.999	(0.969–1.031)	0.967			
Gender Male/female	2.383	(1.086–5.229)	0.030	2.673	(0.989–7.228)	0.053
BMI (kg/m^2^)	0.896	(0.786–1.022)	0.103			
Comorbidity Yes/No	0.808	(0.262–2.494)	0.710			
History of ipsilateral surgery Yes/No	1.739	(0.640–4.726)	0.278			
V-LEU − / + / + + / + + + / + + + +	1.534	(1.062–2.218)	0.023	0.844	(0.465–1.535)	0.579
NIT − / +	3.519	(1.410–8.782)	0.007	3.814	(1.026–14.178)	0.046
Urine culture − / +	1.826	(0.709–4.703)	0.212			
Stone burden (mm^2^)	1.001	(1.001–1.002)	< 0.001	1.001	(1.000–1.002)	0.030
Stone density (HU)	1.000	(0.999–1.001)	0.966			
Degree of hydronephrosis None/mild/moderate/serious	0.933	(0.626–1.393)	0.736			
Stone type Single/multiple/staghorn	1.865	(0.998–3.485)	0.051	0.564	(0.230–1.385)	0.211
Operative time (min)	1.028	(1.016–1.041)	< 0.001	1.020	(1.001–1.040)	0.044
Number of tracts 1/2/3/4–7	2.074	(1.471–2.925)	< 0.001	0.569	(0.274–1.182)	0.131
Tract size Standard/mini	0.262	(0.113–0.607)	0.002	1.000	(0.310–3.227)	0.999
Number of nephrostomy tubes 0/1/2/3/4	3.716	(2.168–6.371)	< 0.001	4.282	(1.779–10.308)	0.008
SFR (%)	2.324	(0.834–6.476)	0.107			

PCNL, percutaneous nephrolithotomy; U-LEU, urine leukocyte; NIT, urine nitrite; SFR, stone free rate; OR, odds ratio; CI, confidence interval

The final multivariate model identified the following independent risk factors for delayed discharge: positive NIT, larger stone burden, longer duration of surgery and multiple nephrostomy tubes. The p-values, odds ratios, and 95% confidence intervals are also presented in Table 2.

### Construction of nomogram for prediction of delayed discharge

**Fig. 1:**
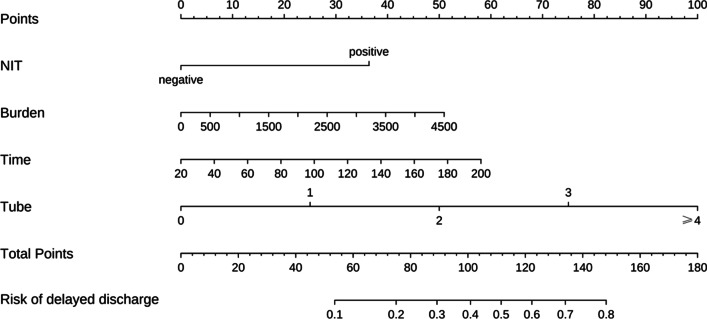
Nomogram for predicting the probability of postoperative delayed discharge after day-surgery PCNL in patients. NIT: Urine nitrite; Burden: Stone burden; Time: Surgery time; Tube: Number of nephrostomy tube. PCNL = percutaneous nephrolithotomy

**Fig. 2:**
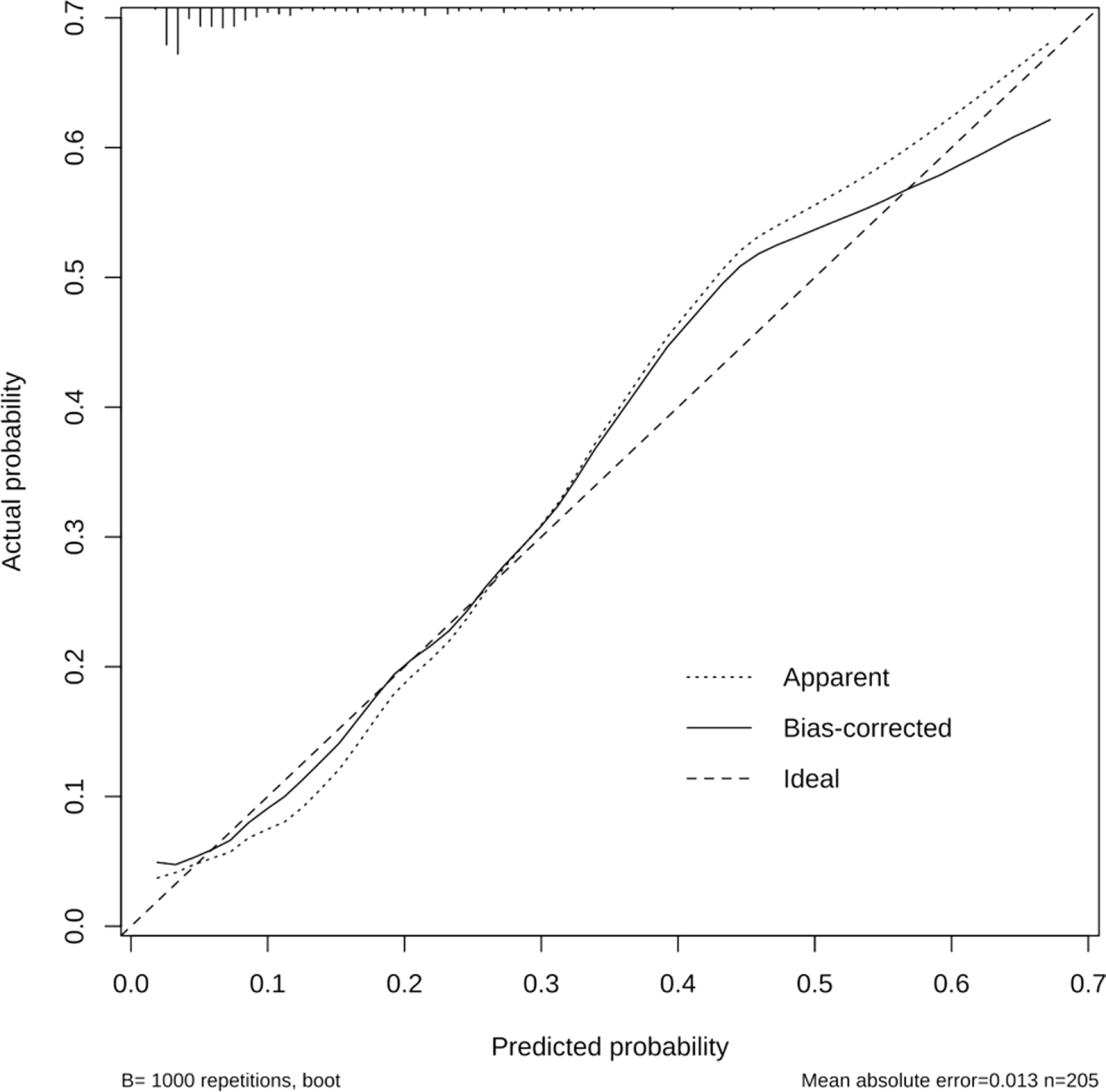
Calibration curve of prediction model for postoperative delayed discharge after day-surgery PCNL in patients. PCNL = percutaneous nephrolithotomy

A nomogram was constructed with four independent predictors based on the result of multivariate analysis (Fig. 1). According to our calculation, the c-index was 0.822. As showed in Fig. 2, the nomogram was well calibrated by the calibration plot.

### Reasons of delayed discharge

A medical reason causing delayed discharge was found in 23 (76.7%) patients. The most frequent reasons included pain (7 patients, 23.3%), bleeding (4 patients, 13.3%), urosepsis or fever (5 patients, 16.7%), and urine leakage (3 patients, 10.0%). psychological reasons accounted for 23.3% of the cases. All of these patients met discharge criteria but refused to discharge because of the fear of the development of complications after discharge. The reasons for delayed discharge are showed in Table 3.

**Table 3:** The reasons for delayed discharge after day-surgery PCNL

Reasons	Case (n = 30, %)
Psychological reasons^a^	7 (23.3%)
Medical reasons	23 (76.7%)
Pain	7 (23.3%)
Bleeding	4 (13.3%)
Urosepsis	3 (10.0%)
Urine leakage	3 (10.0%)
Fever	2 (6.7%)
Pleural effusion	2 (6.7%)
Hypokaliemia	1 (3.3%)
Urinary-tract infection	1 (3.3%)

^a^Psychological reasons were defined as patients encountering discharge criteria but refusing to leave hospital because they feared the development of adverse events

### Follow-up

According to our follow-up, unplanned hospital readmission occurred in 3 (1.5%) patients and no emergency department visit within 30 days after discharge. Six patients who had residual stones underwent second-stage surgery (4 PCNL and 2 ESWL) and 17 patients did not accept further treatment but received regular observations.

## Discussion

LOS, SFR, and severe complications can affect the cost effectiveness of PCNL[15]. Reducing the LOS is a key point to enhance the cost effectiveness of PCNL[5]. Traditionally, hospital stay after PCNL has typically remained 2–5 days[16]. In order to improve patient satisfaction and reduce healthcare costs, urologists have already discharged patients on the same day or overnight after PCNL. However, day-surgery PCNL is not always successful and patients who recover slowly after PCNL may fail to be discharged within 24 h. With the aim of better management of day-surgery patients, this study was designed to identify the reasons and risk factors for delayed discharge after day-surgery PCNL.

We aimed to discharge patients on the same day or within 24 h. Approximately half of the patients who stayed in hospital more than 24 h in our study because of psychological reasons or pain-related issues, which was similar to the results of Seth K. Bechis[6]. Postoperative pain control and perioperative psychological management are crucial for patients to recover quickly. Other medical reasons include bleeding, urosepsis, urine leakage and so on. Early identification of those complications and taking related measures to prevent exacerbation can shorten hospital stay.

To date, few studies have analyzed the risk factors of delayed discharge after day-surgery PCNL. However, some studies have shown that BMI, comorbidities of hypertension or diabetes, and stone size affect the LOS after inpatient PCNL[17–19]. Through logistic regression analysis for the risk factors that were possibly linked to delayed discharge after day-surgery, the results of this study demonstrated that urine nitrite (NIT), stone burden, surgery time and number of nephrostomy tubes had a significant association with delayed discharge.

Based on current knowledge, preoperative NIT has not been reported as an independent risk factor for delayed discharge after day-surgery PCNL. Fan et al. reported that preoperative NIT was an independent predictor for uroseptic shock after minimally invasive PCNL[20]. Another study concluded that preoperative positive NIT might play a more important role than urine culture in the prediction of postoperative fever after retrograde intrarenal surgery[21]. Positive NIT usually warn the existence of UTI[22]. However, positive urine nitrite with a negative urine culture makes it difficult for urological surgeons to select appropriate antibiotics or cause them to ignore anti-infective therapy. According to the current study, patients with preoperative positive NIT were more likely to develop infection-related complications after PCNL, so it was important to select the correct type of antibiotic and ensure an adequate period of usage.

In case of large renal stones, it is often necessary to establish multiple tracts in single session PCNL or secondary PCNL procedures are required to completely remove the stones. Large calculi such as staghorn calculi form over a long period and are usually associated with UTI[23], which will increase the difficulty of surgery. Our study suggests that larger stone burden is an independent risk factor for delayed discharge after day-surgery PCNL, which may be related to the increasing operative time, more operating tracts and more nephrostomy tubes will increase the probability of postoperative complications.

Longer surgery time has, to our knowledge, not previously been associated with an increased possible of deferred discharge. Prolonged surgery time is mostly associated with the difficulty of operation (large stone size, high stone density, complex renal stones), which may increase the risk of postoperative complications such as sepsis and bleeding[24, 25]. This also subsequently hamper the rehabilitation process. Identifying and solving the problems that caused prolonged surgery time could be beneficial in shortening the overall LOS. Furthermore, according to the results of this study, drawn-out surgery time should be regarded as an early warning sign for patients at a higher risk of delayed discharge and who will be therefore paid more attention in the same-day operation unit.

Conventionally, nephrostomy tubes are placed following PCNL to decrease the incidence of postoperative complications, including prevention of urine extravasation, tamponade against possible bleeding, and pledge of enough urine drainage[26, 27]. Moreover, the urological surgeon will have the chance to perform second-stage PCNL through the nephrostomy tube if a residual stone mass is found after surgery. However, the necessity of the nephrostomy tubes is controversial due to its resulting discomfort and morbidity after surgery[28]. Pimentel et al. reported that nephrostomy tube was associated with a longer hospitalization compared to the tubeless technique[29]. According to our analysis, the number of nephrostomy tubes was found to be an independent predictor for delayed discharge after day-surgery PCNL. In our study, placement or not of nephrostomy tubes or how many nephrostomy tubes were placed depended on the following situations: number of channels, a large number of residual stones, obvious channel bleeding, and severe preoperative infection. The reasons that nephrostomy tubes cause delayed discharge can be considered to include postoperative discomfort, catheter-related infection, and the psychology of doctor or the patients, etc. The placing of multiple nephrostomy tubes may mainly be related to large stone burden, multiple puncturing channels, and bleeding in more than two tracts. Under the premise of ensuring safety, tubeless or the use of as few tubes as possible after PCNL can facilitate patient’s recovery.

In addition, the number of nephrostomy tracts was not an independent risk factor for delayed discharge. In a previous study by the current authors, it was concluded that multiple-tract PCNL was an efficient and safe method to treat complex kidney stones[30]. When multiple-tract PCNL is implemented by experienced urologists, it can be considered to reduce the incidence of postoperative adverse events. Multiple-tract PCNL is as safe as single-tract PCNL and does not increase the risk of delayed discharge. However, accurate puncturing must be ensured when performing multiple-tract PCNL because it is a key point to ensure success.

We constructed a nomogram prediction model based on the independent risk factors, including stone burden, NIT, surgery time, and nephrostomy tube. Through internal verification, the c-index of the nomogram was 0.822, indicating good consistency. This model can not only help in clinical decision-making but also provide a visual instrument for postoperative delayed discharge assessment. Taking appropriate measures to address such risk factors can accelerate the recovery of patients after day-surgery PCNL and reduce the rate of delayed discharge.

There were some limitations in this study. First, due to the retrospective nature of this study, there were some biases in the identification of the reasons for deferred discharge after operation. To solve this latent source of bias, the medical records of the patients were scrutinized very carefully to obtain accurate reasons for the delay. Second, the sample size was not large enough as there were only 30 cases of delayed discharge from a total of 205 patients. Lastly, other risk factors such as ASA score, stone composition, etc. were not included. The ASA score of all patients in this cohort study was ≤ 2, so it made no sense to analyze these data. Nevertheless, our study identified some independent risk factors for delayed discharge, which will help urological surgeons to better manage patients in day-surgery PCNL procedures. There is still a need for further multicentric and prospective studies with large sample size to confirm these results.

## Conclusions

The outcomes of our study demonstrated the most frequent reasons for delayed discharge included psychological reasons and pain. Furthermore, four independent risk factors for delayed discharge after day-surgery were identified: larger stone burden, positive urine nitrite, longer duration of surgery, and multiple nephrostomy tubes. Therefore, patients with large renal stones or positive NIT may be at increased risk of delayed discharge. Shortening surgery time and reducing the number of nephrostomy tubes will help to facilitate recovery.

## Data Availability

The datasets used and analysed during the current study are available from the corresponding author on reasonable request.

## Abbreviations

PCNL: Percutaneous nephrolithotomy
U-LEU: Urine leukocyte
NIT: Urine nitrite
LOS: Length of hospital stay
SFR: Stone free ate
ERAS: Enhanced recovery after surgery
UTI: Urinary tract infection
BMI: Body mass index
ASA: American Society of Anesthesiologist
OR: Odds ratio
